# Pre-Operative Combination of Normal BMI with Elevated YKL-40 and Leptin but Lower Adiponectin Level Is Linked to a Higher Risk of Breast Cancer Relapse: A Report of Four-Year Follow-Up Study

**DOI:** 10.3390/jcm9061742

**Published:** 2020-06-04

**Authors:** Kornel Bielawski, Piotr Rhone, Marek Bulsa, Barbara Ruszkowska-Ciastek

**Affiliations:** 1 Department of Pathophysiology, Faculty of Pharmacy, Nicolaus Copernicus University, Collegium Medicum in Bydgoszcz, 85-094 Bydgoszcz, Poland; kornel-bielawski@wp.pl; 2 Clinical Ward of Breast Cancer and Reconstructive Surgery, Oncology Centre Prof. F. Łukaszczyk Memorial Hospital, 85-796 Bydgoszcz, Poland; prhone@wp.pl; 3 Department of Health Sociology and Healthy Behaviours, Faculty of Humanities, University of Szczecin, 71-017 Szczecin, Poland; marekbulsa@wp.pl

**Keywords:** inflammation, adipokines, breast cancer, disease recurrence

## Abstract

Adipokines are powerful agents involved in the development of obesity-dependent cancers. This prospective study aimed to investigate the association between pre-treatment body mass index (BMI) and serum YKL-40, leptin, and adiponectin concentrations as well as the plasma activity of tissue factor (TF) and the future prognosis of early, non-metastatic breast cancer (BrC) subjects. The serum levels of YKL-40, leptin, and adiponectin as well as plasma TF activity, anthropometric parameters, and clinicopathological parameters were analysed in 81 treatment-naïve females with invasive BrC. The predictive value of YKL-40, BMI, leptin, adiponectin, and TF was determined with a 95% confidence interval (CI). Kaplan–Meier plots and log-rank and F Cox tests were used to determine the clinical outcomes of progression-free survival (PFS). The median follow-up duration was 44 months with complete follow-up for the first event. Follow-up revealed a significantly higher incidence of disease relapse in BrC patients with a high baseline concentration of YKL-40 (22.22%) and TF activity (21.43%). Body mass index was an independent predictor of survival, with women who were overweight/obese being less prone to relapse (hazard ratio (HR): 0.75; 95% CI: 0.59 to 0.95). The recurrence rates for normal-weight BrC cases was 21.05% versus 7.14% for their overweight counterparts. The receiver operating characteristic analysis showed the strong ability of the analysed biomarkers to predict disease progression, with an area under the receiver operating characteristics (ROC) curve of 0.84 (95% CI, 0.823 to 0.931). In a prospective cohort of invasive BrC patients, overweight/obesity was associated with improved future outcomes. The combination of a normal BMI with high leptin and low adiponectin levels and high TF activity was associated with an increased risk of recurrence and decreased survival.

## 1. Introduction

It is well-established that breast cancer (BrC) and obesity are the most widespread diseases in the female population, all over the world. There is no doubt that obesity is a well-identified risk factor for cervical, ovarian, endometrial, and breast cancer, and it is associated with 88% mortality rates in females [1,2]. Interestingly, one in two breast cancer women are overweight or obese [2]. Obesity leads to increased susceptibility for postmenopausal luminal breast cancers, but premenopausal obese women are more prone to developing a basal-like breast cancer (BLBC). Furthermore, adiposity is associated with a more aggressive breast cancer phenotype, including larger tumours, more often with nodal metastases and worse prognosis [3,4,5,6]. Thus, adiposity is linked with both cancer predisposition and cancer-dependent death [7].

Against this background, prognostic indicators in patients with breast cancer are fundamental. Since BrC is a very heterogeneous disease with well-defined morphologies, molecular attributes, prognoses, and treatment options, clinical decisions are mainly made on the basis of the tumour stage, lymph-node status, and the expression of molecular determinants, represented clinically by oestrogen receptor (ER), progesterone receptor (PR), human epidermal growth factor receptor 2 (HER2), and the proliferation marker Ki67 [8]. There is a five-year survival rate of 100%, 93%, 72%, and 26% for stages I, II, III, and IV, respectively [9]. Thus, the early diagnosis of breast cancer has great clinical prominence.

Nowadays, the underlying biological mechanisms that link obesity to BrC have largely focused on the excessive exposure of the mammary glands to the abundant secretion of oestrogen, cytokines, pro-inflammatory mediators, and growth factors, along with an altered adipokine profile, all of which are believed to be involved in the promotion, invasion, and metastasis of cancer cells. Normal adipose tissue and mature adipocytes, especially, produce the insulin-sensitising, anti-inflammatory, anti-atherogenic, and anti-proliferative hormone, adiponectin, and a low amount of the pro-angiogenic, pro-mitogenic leptin as well as a low quantity of pro-inflammatory cytokines [3,10]. However, adiposity leads the way in a pivotal shift towards the up-regulation of leptin in parallel to the down-regulation of adiponectin and overproduction of pro-inflammatory M1 macrophages [2,3,11,12].

The additional threat of neoplasm is a pro-coagulant state, in which the enhancement of coagulation is linked to malignant transformation via tumour cell growth, migration, angiogenesis, and extravasation. Cancer-associated overexpression of tissue factor (TF) and thrombin has been noted. Interestingly, TF can also be released under the influence of tumour necrosis factor alpha (TNF-α) and interleukin-1 beta (IL-1β) in soluble form. Thus, these pro-inflammatory agents are incident to the hypercoagulability state. On the other hand, tumour cells produce TNF-α and IL-1β, which activate pro-angiogenic growth factors to cause enhanced tumour growth [7,13]. Summarising, the malignant process, inflammation and the pro-coagulant state are inseparably connected with each other to create the “self-powered machine”.

Chronic low-grade inflammation is a common attribute of adiposity and cardiovascular complications [7,13]. The contributions of the local immune response and systemic inflammation in cancer initiation and invasion have been confirmed [14]. A promising biomarker for predicting the risk of BrC recurrence is an inflammatory protein named YKL-40, also called human cartilage glycoprotein-39 (HC gp-39) and chitinase 3-like 1 protein (CLP). YKL-40 is secreted by leukocytes, tumour cells, and tumour-related macrophages. Il-6 and hypoxia induce YKL-40 synthesis [14,15,16,17,18]. The serum and plasma levels of YKL-40 are associated with poor clinical outcomes in breast, lung, prostate, liver, bladder, colon, and other types of tumours [14,15,16,17,18]. YKL-40 prevents apoptosis and promotes malignant cell proliferation by the up-regulation of anti-apoptotic or pro-proliferative, pro-angiogenic factors. In severe infections, increases in plasma YKL-40 are noted before rises in the plasma C-reactive protein [18]. Interestingly, Kjaergaard et al. suggest that plasma YKL-40 is not a causal agent of neoplastic changes but rather an inflammatory biomarker [14].

It is crucial to recognise the association between adiposity and the activation of TF-dependent coagulation and inflammatory reactions and to elucidate their importance in malignant transformation. Thus, the purpose of the present study was to assess the association between pre-treatment body mass index (BMI), serum YKL-40, leptin, adiponectin concentrations, the plasma activity of TF, and the future prognosis for early, non-metastatic BrC subjects. 

## 2. Material and Methods

### 2.1. Compliance with Ethical Standards

Written informed consent was received from all patients in accordance with the Declaration of Helsinki. Subjects could withdraw from the study before or at any time during the study. The present research protocol was approved by the local bioethical committee (permission no. KB 547/2015; 16.06.2015).

### 2.2. Description of the Patients’ Cohort

Eighty-one consecutive female subjects with newly diagnosed, histologically-confirmed invasive BrC were enrolled in this study. The median age at inclusion was 54 years (interquartile range, 49–59 years). The participants were enrolled in the study before systemic adjuvant treatment. Since the levels of adipokines and pro-inflammatory markers may vary with advancement of disease, only patients with Stage I and II disease (according to the AJCC classification of 2016) were included in the present series (Stage I, *n* = 40; Stage II, *n* = 41). The patients were under the care of the medical staff from the Clinical Ward of Breast Cancer and Reconstructive Surgery, Oncology Center Prof. F. Łukaszczyk Memorial Hospital, Bydgoszcz, Poland.

### 2.3. Inclusion and Exclusion Criteria

The inclusion criteria of the study were as follows: (1) the diagnosis of primary, unilateral, invasive BrC; (2) complete clinical record and follow-up data; (3) all the peripheral blood samples were obtained within 24 h before surgery; and (4) proper haematological, liver, and renal function. The exclusion criteria for the patients included: (1) bilateral BrC; (2) a tumour larger than 5 cm; (3) Stage IIIA or higher; (4) neoadjuvant chemotherapy, radiotherapy, or endocrine therapy; (5) a previous diagnosis of any cancer type; (6) distant metastasis; (7) chronic inflammatory diseases or autoimmune disease; (8) a carcinoma in situ; (9) morbid obesity (BMI over 40 kg/m^2^); (10) diabetes mellitus type 2; (11) psychiatric illness; and (12) male gender.

### 2.4. Collection of Clinical Data

All of the study participants underwent a personal interview administered by oncologists in the hospital. Data were collected on the sociodemographic characteristics (such as age and education), menstrual and reproductive history, menopausal status, menopausal hormone therapy, lifestyle behaviours, and medical history as well as the history of breast and other cancers using a standardised lifestyle questionnaire. Postmenopausal status was identified in the current study as the absence of a menstrual cycle for 12 months after the last menstruation. Body mass index (BMI) (weight in kg/height in m^2^) was calculated from the patient’s height and weight, measured at the initial patient visit, when the individuals were wearing no shoes and few clothes. Tumour histology and size; lymph-node status; tumour staging; and immunohistochemistry (IHC) of the oestrogen receptor (ER), progesterone receptor (PR), human epidermal growth factor receptor 2 (HER-2), Ki67- proliferation marker, and E-cadherin were determined. The Nottingham histological grade of malignancy (the Elston-Ellis modification of the Scarff-Bloom-Richardson grading system) was established, based on three components: (1) the amount of tubule formation, (2) the nuclear grade, and (3) the mitotic rate. The tumour size was defined by the maximum diameter of the sample. The tumor (T)/node (N)/metastasis (M) stage of the disease at the initial diagnosis was confirmed by the American Joint Committee on Cancer (AJCC), 7th Edition. The histological type was classified as ductal carcinoma or lobular carcinoma according to the WHO Classification of Tumours. The intrinsic subtypes of BrC were divided into luminal A, luminal B HER2-positive, luminal B HER2-negative, non-luminal HER2-positive, and basal-like.

### 2.5. Follow-Up Details

All the included patients had post-operative inpatient or outpatient follow-up every three months for the first and second years, and every six months for the third and fourth years after surgery. For the progression-free survival analysis, 11 events were recorded, including 3 (3.7%) loco-regional recurrences, 3 (3.7%) distant metastases, and 5 (6.17%) deaths. The median follow-up time after the index date was 44 months with a 13.58% recurrence rate. Follow-up evaluation included laboratory tests (blood biochemical test), physical examination (breast and lymph-node palpation), breast ultrasonography, liver ultrasound, mammography, and other suitable examinations. Relapse was defined as signs of metastatic disease or local recurrence confirmed by PET/CT or death (excluding deaths unrelated to the disease).

### 2.6. Blood Sampling and Laboratory Tests

Venous blood (4.5 mL) for testing TF activity was collected into cooled tubes (Becton Dickinson Vacutainer^®^ System, Plymouth, UK) containing 0.13 mol/L of trisodium citrate (final blood anticoagulant ratio, 9:1). Additionally, the blood was collected into 4.0 mL tubes (Becton Dickinson Vacutainer^®^ System, Plymouth, UK) without anticoagulant in order to determine the concentrations of adiponectin, leptin, and YKL-40. Blood samples from all patients were obtained within 24 h before surgery procedures, when patients had been in a fasting state; after 30 min of rest and after a 12 h overnight fast, blood sampling took place between 7.00 a.m. and 9.00 a.m., to minimise diurnal variability. The samples were processed according to the standard conditions for blood samples. All clinicopathological data were obtained as part of routine care. All the blood samples were stored at −80 °C until analysis. The blood samples were centrifuged at 3000× *g* for 15 min. There was no variation in the average storage time between the case samples. For all kits, the reaction mixture was added to in a 96-well plate. The laboratory assistants were blinded to the study population.

### 2.7. Serum Leptin Measurement

The baseline serum leptin concentration was measured using a commercially available kit—Human Leptin Enzyme-Linked Immunosorbent Assay (ELISA) Clinical Range (BioVendor Research and Diagnostic products, Brno, Czech Republic; catalogue number: RD191001100)—in accordance with the manufacturer’s instructions. The limit of leptin detection was 0.2 ng/mL. The intra-assay coefficient of variation (within-run) was 5.9% with an inter-assay coefficient of variation (run-to-run) of 5.6%. The subjects were separated as having low or high values, dichotomised using a cut-off for leptin of 12.58 ng/mL, based on the median value for the whole study population.

### 2.8. Serum Adiponectin Analysis

The pre-treatment serum adiponectin level was determined by a human adiponectin ELISA high sensitivity ELISA kit, (BioVendor Research and Diagnostic products, Brno, Czech Republic; catalogue number: RD191023100). The limit of adiponectin detection was 0.47 ng/mL and had an intra-assay coefficient of variation (within-run) within 3.9% and an inter-assay coefficient of variation (run-to-run) of 6.0%. Patients were divided as having low or high values, dichotomised using a cut-off for adiponectin of 27.05 ng/mL, based on the median for the whole study population.

### 2.9. Serum YKL-40 Assay

The baseline serum YKL-40 protein concentration was assessed with a Human Chitinase 3-like 1 ELISA kit (BioVendor Research and Diagnostic products, Brno, Czech Republic; catalogue number: RD193444200CS). The minimal detectable limit for YKL-40 was 10 ng/mL. The intra-assay coefficient of variation (CV) was 5%, and the inter-assay CV was <6%. Patients were grouped as having low or high values, dichotomised using a cut-off for YKL-40 of 1.93 ng/mL, based on the median value for the whole study population. Additionally, the YKL-40 concentration was categorised either as low (<1.60 ng/mL), moderate (1.60–2.50 ng/mL), or high (>2.50 ng/mL) based on the assumption to create three numerically equal subgroups; each group consisted of 27 cases.

### 2.10. Citrate Plasma TF Activity

The plasma activity of TF was measured using chromogenic assays, the ACTICHROME^®^TF test (Sekisui Diagnostics, LLC, Stamford, CT, USA; catalogue number: 846). The minimal detectable limit for TF activity was 2 pM. The standard range was set at 0–30 pM. Patients were grouped as having low or high values, dichotomised using a cut-off for TF of 14.18 pM, based on the median value for the whole study population. Additionally, TF activity was categorised either as low (<12 pM), moderate (12–24 pM), or high (>24 pM) based on the assumption to create three equal subgroups; the first group consisted of 26 cases, the second was made up of 27 patients, and the third was composed of 28 subjects (there were 28 patients in the third group due to the fact that two patients had the same results).

### 2.11. Immunohistochemical Detection of Hormone Receptors, Ki67, and HER2

The measurements of hormone receptors including ER and PR as well as HER2 and Ki67 were performed by the immunohistochemistry method according to the manufacturer’s indications. To define ER and PR status, immunostaining using anti-ER (clone SP1) and anti-PR (clone 1E2) pre-diluted monoclonal antibodies (Ventana Medical Systems, Tucson, AZ, USA) was applied. For the demonstration of the Ki67 antigen in the specimens, monoclonal mouse antibody (Autostainer Link 48, Agilent Technologies, Santa Clara, CA, USA) was used. For the semi-quantitative detection of HER2, the rabbit monoclonal primary antibody VENTANA anti-HER2/neu (clone 4B5) was used.

The immunostaining for ER, PR, and Ki67 was quantified and expressed as percentages of immunostained neoplastic cells. We used 20% as the threshold value to judge the level of Ki67 expression. For HER2, strong complete membrane staining in more than 10% of tumour cells was defined as positive expression (3+). Scores of 0 and 1 were established as negative expression, and all tumour cells with a 2+ score were further tested by fluorescence in situ hybridisation [19]. Based on the expression of molecular determinants, all subjects were classified according to the St. Gallen 2013-15 recommendations.

### 2.12. Statistical Methods

The Shapiro–Wilk test was used to test normality, and the Mann–Whitney test and the ANOVA Kruskal–Wallis test were used to compare subgroups. The non-normally distributed variables were described by medians and interquartile ranges (IQR). The clinicopathologic categorical variables were presented as numbers and percentages (%). The Kaplan–Meier product limit estimator method was used to determine the survival time of PFS. The log-rank test and the F Cox test were used to check the dependence of patients’ survival on single or on combinations of variables. The multivariate Cox proportional hazards regression model was used to estimate the hazard ratios (HRs) and the 95% confidence intervals (CIs) for the associations between the serum YKL-40, leptin, and adiponectin levels as wells as the plasma TF activity and the progression-free survival. A 95% confidence interval (95% CI) was set as the criterion to establish statistical significance. Finally, to evaluate the biomarkers’ ability to predict disease recurrence, analysis of the receiver operating characteristics curve (ROC) by the area under the curve (AUC) was performed according to the logistic regression model. Differences with *p*-values of less than 0.05 were considered to be statistically significant.

## 3. Results

### 3.1. Clinical Summarisation of the Study Population

Between November 2015 and January 2018, from a total of 92 newly diagnosed and treatment-naïve BrC subjects, 81 patients fulfilled the inclusion/exclusion criteria and were prospectively included in the study. Table 1, Table 2 and Table 3 summarise the study group according to anthropometric, demographic, and clinicopathological features as well as biochemical parameters. All cases were females, and the median age was 54 years (interquartile range (IQR): 49–59 years). The median BMI was 25.22 kg/m^2^ (IQR: 23.05–28.90 kg/m^2^). More than half (53%) of the cases were overweight or obese. The median follow-up for the entire cohort was 44 (IQR: 37–48) months, with complete data for the cause and date of death. All 81 patients underwent surgery, 13 cases underwent mastectomy, and 68 cases underwent breast-conserving surgery. In terms of the histological pathology, 69 patients had invasive ductal carcinoma (IDC) and 12 patients had invasive lobular carcinoma (ILC). Fifty (62%) BrC subjects had a luminal-A intrinsic subtype. The most common histological grades according to Elston–Ellis classification were highly (G1) and moderately differentiated carcinomas G2 (80%). The median tumour diameter was 1.67 cm (IQR: 1.2–2.1 cm). More than the 75% of cases were free of nodal involvement.

### 3.2. Analysed Parameters According to Age, Menopausal Status, Parity Status, and Body Mass Index among BrC Cases

The age at diagnosis was categorised into two groups (<55/≥55 years) as well as menopausal status (premenopausal/postmenopausal). A lack of significant differences in the concentrations of YKL-40, leptin, and adiponectin as well as the TF activity with respect to age and menopausal status was noted. BMI (kg/m^2^) was classified as normal (18.5–24.9), overweight (25.0–29.9), and obesity class I and II (30.0–39.9) according to the WHO’s recommendations. Parity was defined as the number of full-term pregnancies. There was a trend towards a higher concentration of leptin and a lower concentration of adiponectin with increasing BMI in our study group. Interestingly, the concentrations of adiponectin and YKL-40 were dependent on parity status. A significantly higher concentration of YKL-40 was observed in BrC subjects who gave birth to one child than in cases who gave birth to two or three children. Patients who gave birth to one child or two children had a significantly higher concentration of adiponectin compared to subjects who gave birth to three children (Table 1).

### 3.3. Distribution of YKL-40, Leptin, and Adiponectin Concentrations and TF Activity According to Immunohistochemical Determinants in the Study Cohort

In Table 2, the statistical calculations for the study group are presented based on the proliferation marker (Ki67), human epidermal growth factor receptor 2 (HER2), and the status of hormone receptors with respect to the analysed parameters. The patients were divided into two groups on the basis of the proliferation marker, expressed by the Ki67 protein. The first group was made up of women with expression of the Ki67 antigen lower than 20%, and the second group included patients with Ki67 expression above 20%. A markedly higher concentration of adiponectin was observed in patients with expression of Ki67 lower than 20% (*p* < 0.01). Other molecular determinants did not differ significantly with regard to YKL-40, leptin, adiponectin, and TF activity.

### 3.4. Distribution of YKL-40, TF Activity, Leptin, and Adiponectin According to Clinicopathological Characteristics in the Study Group

Furthermore, we hypothesised that the concentrations of YKL-40, leptin, and adiponectin and TF activity can vary according to the molecular subtype, tumour diameter, nodal status, staging, grading indexing, and histological subtypes of BrC. A significantly higher concentration of adiponectin in the luminal A-type of BrC compared to patients having a basal-like type was noted (*p* < 0.01). Additionally, a significantly higher TF activity was observed in the luminal B HER2-negative type of BrC with respect to patients having other molecular subtypes, including luminal A, luminal B HER(+), and HER(+)/non-luminal, basal-like BrC (*p* < 0.01). Interestingly, we observed significantly higher concentrations of YKL-40 and leptin (both *p* < 0.01), but a lower concentration of adiponectin (*p* < 0.001) in the group of patients with invasive ductal carcinoma with respect to their invasive lobular carcinoma counterparts. Furthermore, circulating YKL-40, leptin, and adiponectin concentrations and TF activity have not been associated with other prognostic indicators, such as tumour grade and TNM stage or tumour size and nodal status (Table 3).

### 3.5. Predictive Factors for Invasive BrC in Multivariate Cox Proportional Hazards Regression Analysis

The multivariate Cox hazards analysis, which takes into account the function of time, revealed that the activity of tissue factor (HR, 1.07; 95% confidence interval (CI), 1.01–1.14; *p* = 0.0240), leptin concentration (HR, 1.21; 95% CI, 1.02–1.44; *p* = 0.0327), BMI (HR, 0.75; 95% CI, 0.59–0.95; *p* = 0.0178), and adiponectin level (HR, 0.86; 95% CI, 0.76–0.97; *p* = 0.0135) were significant predictive factors for disease relapse in BrC subjects. The multivariate Cox regression showed that BrC subjects with lower BMI values and adiponectin levels but with higher TF activity and leptin concentrations have a significantly higher risk of recurrent events (Table 4).

### 3.6. Assessment of the Analysed Parameters’ Ability to Predict Disease Recurrence; Analysis by the Receiver Operating Characteristics Curve (ROC)

**Figure 1:**
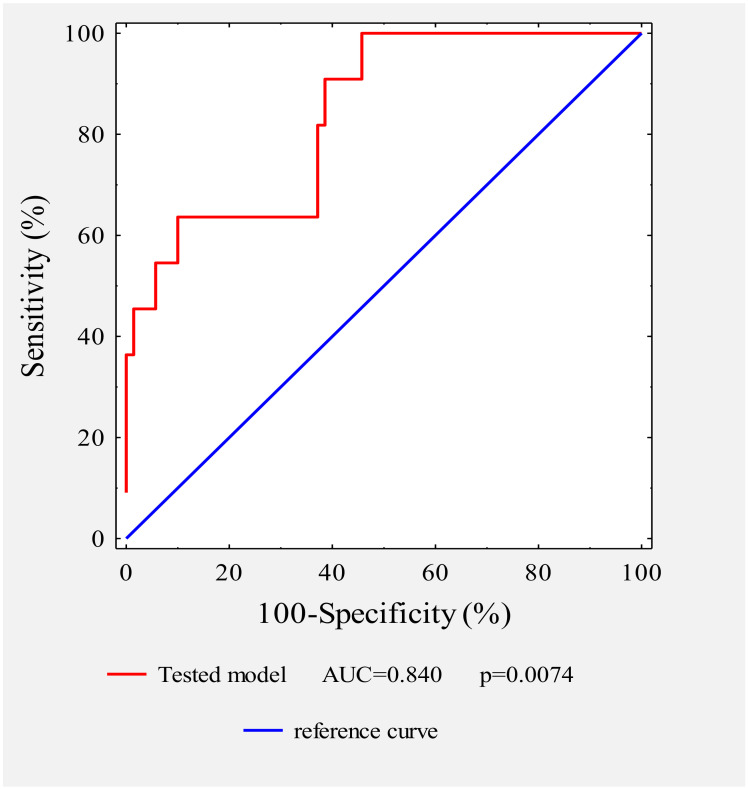
Receiver operating characteristic (ROC) curve for the tested model in patients with versus without disease progression. The area under the curve (AUC) and the *p* value are indicated.

Additionally, we performed receiver operating characteristic (ROC) curve analysis according to logistic regression to describe the ability of the five biomarkers including BMI and TF activity and the concentrations of YKL-40, leptin, and adiponectin to predict disease relapse (Figure 1). The estimated area under the ROC curve for the tested model was AUC = 0.84, with 95% CI = 0.823 to 0.931, R^2^ = 0.7515, and *p* value = 0.0075. This model points to strong diagnostic potential for the prediction of disease progression.

### 3.7. Survival Predictive Value of Pre-Treatment YKL-40 Concentration (A), TF Activity (B), and BMI (C)

**Figure 2:**
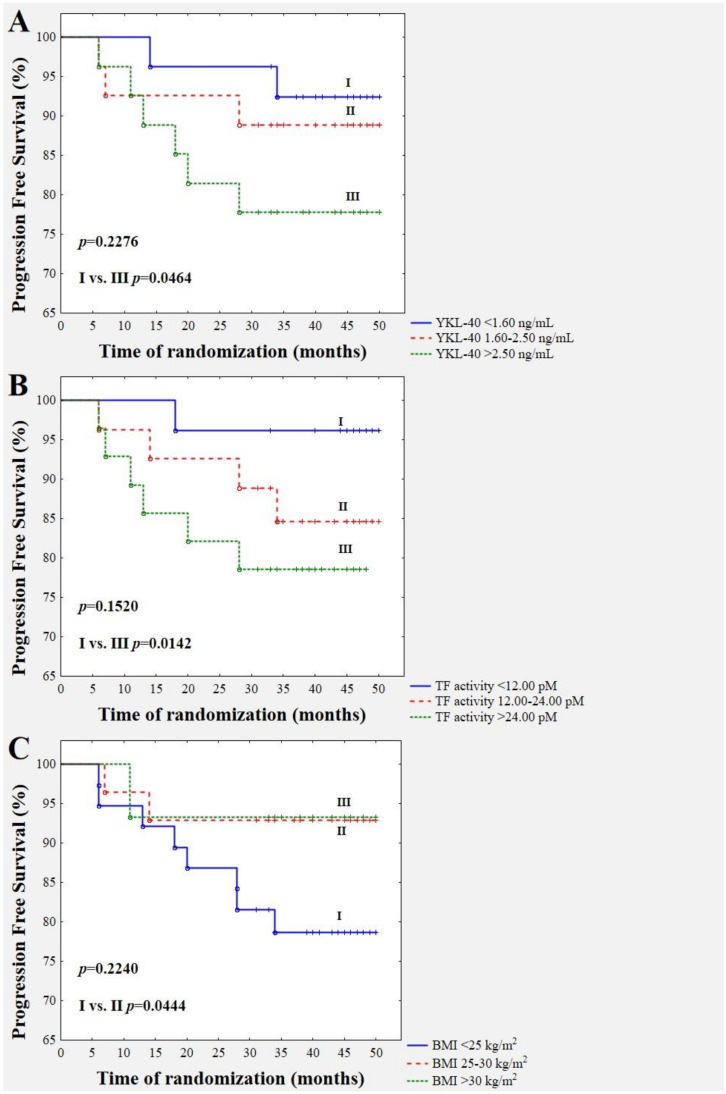
Kaplan–Meier survival curves showing baseline serum YKL-40 (**A**), TF activity (**B**), and BMI (**C**), divided according to cut-offs for YKL-40: < 1.6 ng/mL, 1.6–2.5 ng/mL, and >2.5 ng/mL; TF activity: <12 pM, 12–24 pM, and >24 pM; and BMI value: <24.9 kg/m^2^, 25–29.9 kg/m^2^, and >30 kg/m^2^, respectively.

We analysed the association between the pre-treatment concentrations of YKL-40, leptin, and adiponectin and TF activity as well as body mass index and disease relapse based on the 44 month follow-up of those patients (Figure 2). The data show that, during the follow-up period of more than four years, 11 of the 81 BrC patients (13.58%) had a relapse of disease. Five (6.17%) patients died during the follow-up period due to systemic metastatic disease (bones, liver, and spine metastases). Figure 2A demonstrates the division of the study group according to YKL-40 concentration. In the low YKL-40 subgroup, recurrent events occurred in two subjects out of the total of 27 followed-up subjects (7.41%). In the moderate YKL-40 subgroup, recurrent events happened in three patients out of the total of 27 followed-up subjects (11.11%). Finally, in the high YKL-40 subgroup, recurrent events occurred in six cases out of the total of 27 followed-up subjects (22.22%). Thus, patients with YKL-40 higher than 2.5 ng/mL demonstrated a significantly higher risk of disease recurrence compared to patients with a YKL-40 concentration lower than 1.6 ng/mL (*p* = 0.0464). Patients with TF activity lower than 12 pM (*n* = 26) had a significantly lower risk of disease progression (3.85%) with respect to those patients with TF activity higher than 24 pM (21.43%; *n* = 28); *p* = 0.0142 (Figure 2B). Furthermore, the study group was divided into three subgroups according to BMI values. There was a significantly higher incidence of disease relapse in BrC patients with a BMI (*n* = 38) lower than 24.9 kg/m^2^ compared to those with a BMI of 25–29.9 kg/m^2^ (*n* = 28; *p* = 0.0444). The recurrence rate for normal-weight BrC cases was 21.05% versus 7.14% for overweight cases. Thus, a BMI lower than 25 kg/m^2^ was associated with a three-fold higher risk of disease relapse (Figure 2C). In those eight normal-weight recurrent cases (the mean value of BMI was 22.4 kg/m^2^), three of them had luminal A, three had luminal B HER-, and two had a basal-like intrinsic subtype of BrC. Four patients were premenopausal, and four of them were postmenopausal. All of the patients were symptomatically free of other co-morbidities. All of them had moderately differentiated carcinomas (G2). Two out of eight had a positive lymph-node status. Four out of eight had overexpression of the proliferation marker Ki67. Five out of eight had a tumour larger than 2 cm. Five out of eight underwent breast-conserving surgery due to a lack of lymph node metastases. Additionally, five out of eight received radiotherapy, chemotherapy, or hormone therapy. According to additional analysis in those eight normal-weight recurrent women, the highest level of YKL-40 and TF activity but the lowest adiponectin level were observed (Table 5). Due to the limited space in the manuscript, we do not show separate plots for leptin and adiponectin, since neither demonstrated significant associations with disease recurrence.

### 3.8. Survival Time Analysis with Respect to the Combination of YKL-40 Concentration with TF Activity (3A), the BMI Value with TF Activity (3B), the BMI Value with YKL-40 Level (3C), the BMI Value with Leptin Level (4A), and the BMI Value with Adiponectin Level (4B)

**Figure 3:**
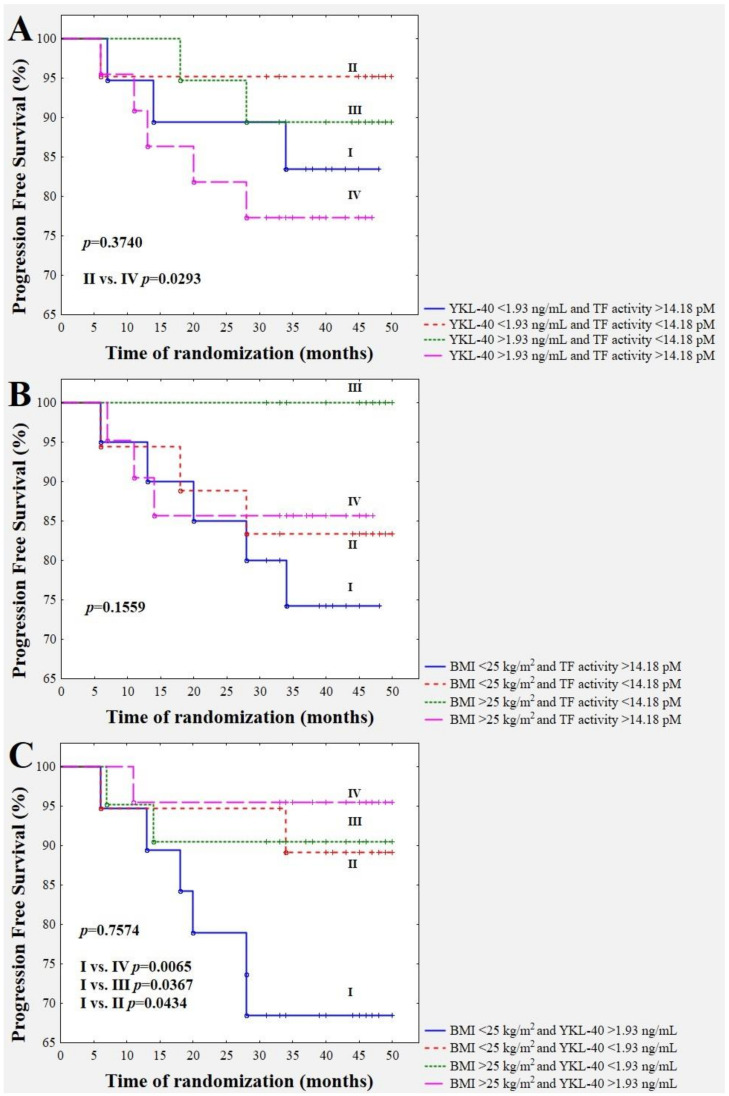
Combination of the YKL-40 level with TF activity (**A**), the value of BMI with YKL-40 (**B**), and BMI with TF activity (**C**), in disease relapse prediction.

**Figure 4:**
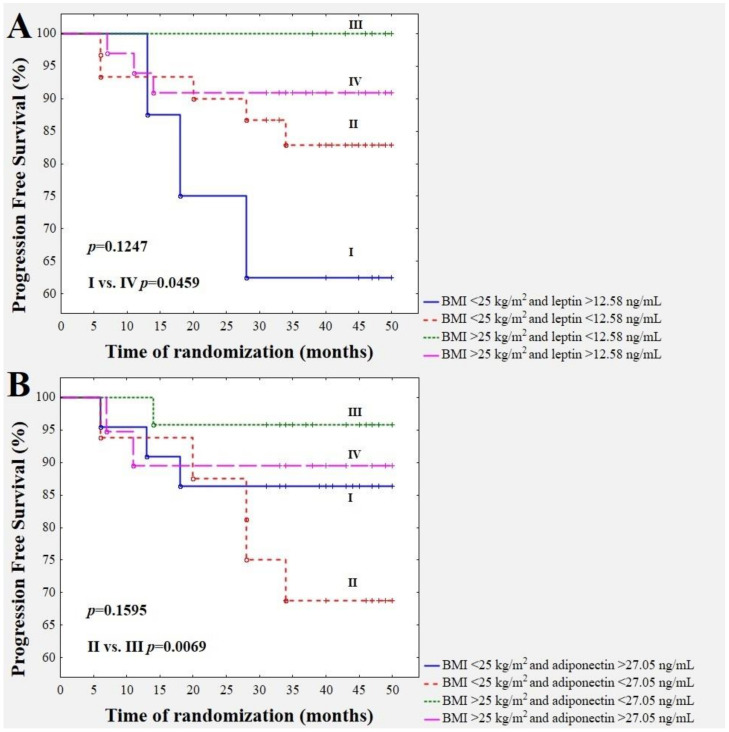
Combination of BMI with the leptin concentration (**A**) and the combination of BMI with the adiponectin concentration (**B**) with respect to disease relapse prediction.

A combination of YKL-40 concentration with TF activity in BrC cases revealed an interesting observation. A worse future prognosis was shown in subjects with a combination of a YKL-40 concentration higher than 1.93 mg/mL and a TF activity higher than 14.18 pM (Figure 3A). Figure 3C and Figure 4A demonstrate a significantly better BrC specific survival for overweight/obese subjects with higher YKL-40 (>1.93 ng/mL) and higher leptin (>12.58 ng/mL) levels with respect to normal-weight cases with YKL-40 higher than 1.93 ng/mL and leptin higher than 12.58 ng/mL (*p* = 0.0065 and *p* = 0.0459, respectively). The recurrence of the disease in the group of overweight/obese patients with higher YKL-40 (>1.93 ng/mL) occurred in one out of 22 (4.55%) cases, but in the subgroup with normal weight and higher YKL-40 levels (>1.93 ng/mL), six out of 19 (31.58%) cases had a recurrence of the disease. The recurrence of the disease in the group of overweight/obese patients with a leptin concentration higher than 12.58 ng/mL occurred in three out of 33 (9.09%), but in the subgroup with normal weight and leptin levels higher than 12.58 ng/mL, three out of eight (37.5%) cases had a recurrence of the disease. Furthermore, follow-up revealed a significantly higher incidence of disease relapse in breast cancer patients with a BMI lower than 25 kg/m^2^ and an adiponectin level lower than 27.05 ng/mL with respect to those with a BMI higher than 25 kg/m^2^ and an adiponectin level lower than 27.05 ng/mL (*p* = 0.0069) (Figure 4B). The recurrence of the disease in the group of patients with a BMI lower than 25 kg/m^2^ and an adiponectin level lower than 27.05 ng/mL occurred in five out of 16 (31.25%), but in the group with a BMI higher than 25 kg/m^2^ and an adiponectin level lower than 27.05 ng/mL, only one out of 24 (4.17%) cases had a recurrence of the disease.

### 3.9. Survival Time Analysis with Respect to Parity Status

**Figure 5:**
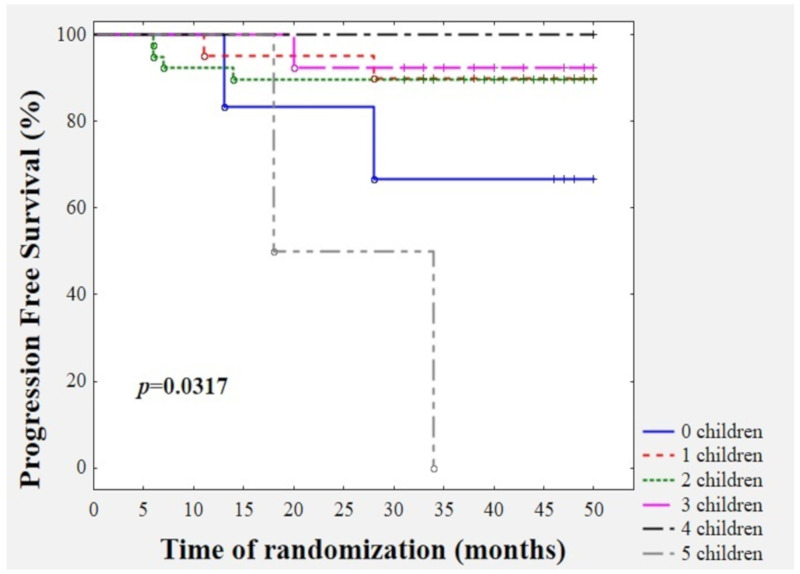
Kaplan–Meier survival analysis according to parity status.

According to our invasive breast cancer cohort, we demonstrated that parity status may predict disease recurrence (*p* = 0.0317). Interestingly, nulliparous women (33.3%) as well as women who gave birth to five children (100%) had the worst future prognosis (Figure 5). Both nulliparous women were postmenopausal subjects, free of other co-morbidities, with BMI = 24 kg/m^2^ and 22.41 kg/m^2^, and age = 55 and 60 years, respectively. The first patient had the following clinicopathological profile: IIA, T2, N0, M0, invasive ductal carcinoma, histological grade = 2, tumour diameter = 2.2 cm, ER+/PR- and HER2-, Ki-67 = 20%, and luminal B HER2 (-). The baseline values were YKL-40 = 1.99 ng/mL, leptin = 34.1 ng/mL, adiponectin = 20.51 ng/mL, and TF activity = 14.10 pM. The patient, after 28 months of follow-up, developed right ovary metastasis. The second patient presented the following clinicopathological profile: IIB, T2, N1, M0, invasive ductal carcinoma, histological grade = 2, tumour diameter = 2.8 cm, ER+/PR+ and HER2-, Ki-67 = 80%, and luminal B HER2 (-). The pre-treatment values were YKL-40 = 2.83 ng/mL, leptin = 14.41 ng/mL, adiponectin = 34.83 ng/mL, and TF activity = 39.16 pM. After 13 months of follow-up, multi-organ metastases were confirmed by PET. Both multiparous women (five children) were premenopausal subjects, free of other co-morbidities with BMI = 21.97 kg/m^2^ and 24.24 kg/m^2^, and age = 49 and 53 years, respectively. The first patient demonstrated the following clinicopathological profile: IIA, T2, N0, M0, invasive ductal carcinoma, histological grade = 2, tumour diameter = 2.5 cm, ER+/PR+ and HER2-, Ki-67 = 40%, and luminal B HER2 (-). The baseline values were YKL-40 = 1.36 ng/mL, leptin = 4.1 ng/mL, adiponectin = 14.58 ng/mL, and TF activity = 23.59 pM. The patient, after 34 months of follow-up, developed bone, mediastinal, and supraclavicular lymph-node metastases confirmed by PET. The second patient showed the following clinicopathological profile: IIB, T2, N1, M0, invasive ductal carcinoma, histological grade = 2, tumour diameter = 3.0 cm, ER+/PR+ and HER2-, Ki-67 = 15%, and luminal A. The pre-treatment values were YKL-40 = 2.55 ng/mL, leptin = 16.92 ng/mL, adiponectin = 28.49 ng/mL, and TF activity = 8.05 pM. However, this patient passed away due to lung, liver, and bone metastases within 18 months of diagnosis.

## 4. Discussion

BrC is a complex neoplastic disease, comprising the processes of unsuppressed proliferation, the elusion of growth suppressors, abnormalities in cell death, the promotion of a pro-angiogenic phenotype, the initiation of invasion and metastasis, the disruption of cellular homeostasis, and the avoidance of immune destruction with the additional significant possibility of relapse [11,20]. Thus, the complex heterogeneity of breast cancer at the molecular level forces a search for prognostic biomarkers, which can be simply estimated or implemented.

### 4.1. Baseline Values of YKL-40, Leptin, Adiponectin, and TF Activity with Respect to Demographic, Anthropometric, and Clinicopathological Features

Adipokines and coagulation factors as well as pro-inflammatory cytokines, have been proposed as a link between obesity and oncogenesis and are related to future prognosis. Our analyses showed a significantly higher concentration of leptin as well as a lower concentration of adiponectin with increasing BMI in breast cancer subjects. This configuration of circulating adipokines is widely accepted in the literature, since body composition changes with age, especially the amount of body fat, which leads to the attenuation of energy homeostasis and variation in adipose tissue metabolism [2,3,11,21,22]. Leptin and adiponectin in cancer biology work in an inverse manner, since leptin plays a crucial role in the promotion of tumour angiogenesis, cell invasion, and metastasis, whereas adiponectin possesses anti-tumour properties, including anti-proliferative, anti-migratory, and pro-apoptotic actions [2,3,21]. Our findings are in line with previous studies, which demonstrated that leptin positively correlates with breast cancer risk, while a low adiponectin concentration is linked to a higher risk of breast cancer [23]. The overexpression of leptin and down-regulation of adiponectin are most commonly associated with progressing or poor prognosis tumours [21,24].

Interestingly, in our study, we observed that the concentration of YKL-40 and adiponectin levels vary with respect to parity. A significant higher concentration of YKL-40 was observed in breast cancer subjects who gave birth to one child than in those cases who gave birth to two or three children. Patients who gave birth to one child or two children had a significantly higher concentration of adiponectin compared to subjects who gave birth to three children. Due to the fact that adiponectin is recognised as an anti-cancer protein, its higher concentration seems to have a protective effect in those patients [3,10]. However, a higher concentration of YKL-40 in those cases can attenuate this positive effect, since YKL-40 is a pro-cancerogenic, pro-angiogenic, pro-inflammatory protein [14,15,16,17,18]. Furthermore, nulliparous as well as multiparous (five children) BrC subjects had the worst future prognosis. According to our results, we speculate that nulliparous and multiparous BrC patients developed a more aggressive phenotype of breast cancer, expressed by a larger tumour size, a higher stage, and a higher expression of the mitotic index. Interestingly, all of those patients had a BMI in the normal range and three out of four had a concentration of YKL-40 higher than 1.93 ng/mL. Three of those four patients demonstrated the luminal B HER2-negative type of BrC.

The present study also shows that the adiponectin level is higher in breast cancer cases with a lower mitotic index, expressed by Ki67, compared to cancers with overexpression of Ki67. Ki67 is a proliferation marker in clinical practice as a marker of tumour aggressiveness and a predictor of future outcomes. Additionally, a higher adiponectin level was observed in luminal A breast cancer cases than in their basal-like counterparts. The luminal A breast cancer type is defined as hormone receptor-positive and HER2-negative with an expression of Ki67 lower than 20%. Thus, our study confirmed previous findings that the adiponectin concentration depends on the oestrogen receptor status [25,26]. Those observations indicate an inverse association between the adiponectin concentration and an aggressive phenotype, and the adiponectin level depends more on the molecular status than clinical features, including tumour diameter or lymph-node status. It is well-known that luminal A breast cancer presents a better prognosis than other intrinsic subtypes. Adiponectin diminishes proliferation, stimulates apoptosis, and negatively influences breast carcinogenesis and the ability for invasion and metastasis [27]. This result may also confirm that luminal A breast cancer with low expression of Ki67 presents the possibility of a more favourable outcome for patients in light of the anti-neoplastic properties of adiponectin. However, the findings of Ando et al. and Panno et al. demonstrated that adiponectin in ER-negative BrC cells diminishes cell growth and suppresses proliferation, invasion, and cancer cell motility [25], but divergent effects of adiponectin were noted with respect to luminal-like BrC cells [26]. A scientific consensus with respect to adiponectin’s role in neoplastic diseases is needed; thus, we would recommend analysis of adiponectin levels in the context of hormone receptor status in subsequent studies. Furthermore, significantly higher TF activity was observed in the luminal B HER2-negative type of breast cancer with respect to patients having other molecular subtypes. The overexpression of TF is associated with an invasive character of tumours and may be particularly related to cancer-associated thrombosis. Moreover, TF stimulates an *angiogenic switch*, leading to new vessel formation and further cancer cell growth [28]. Thus, elevated TF activity is associated with decreased overall survival [28,29]. TF activity and adiponectin levels are sensitive to the tumour’s molecular status and may serve as single biomarkers for future prognosis.

Interestingly, we observed significantly higher concentrations of YKL-40 and leptin but a lower concentration of adiponectin in the group of patients with invasive ductal carcinoma with respect to their invasive lobular carcinoma counterparts. This constellation of cytokines seems to be unfavourable for those patients due to the role of leptin in the production of anti-apoptotic agents, pro-inflammatory cytokines (TNF-α, IL-6), growth factors (VEGF), and the hypoxia-inducible factor-1a (HIF-1α), promoting the cancerous behaviour of cells [23]. Additionally, leptin has been shown to be a potential prognostic marker in breast cancer, along with tumour size and lymph-node status, and an independent predictor of a poor outcome [22]. Additionally, YKL-40 is a key pro-inflammatory cytokine associated with the pathology of obesity-linked cancers. A high YKL-40 serum concentration was significantly associated with invasive lobular carcinoma, TMN stage III, lymph-node metastases, and death [17]. However, a low adiponectin concentration is associated with a more aggressive BrC character [2,10].

### 4.2. YKL-40 Level and TF Activity as Predictors of Disease Recurrence

Our analysis indicates that the pre-treatment concentration of YKL-40 and TF activity are suitable prognostic biomarkers of progression-free survival (PFS) with respect to considering them as a single parameter as well as combining them. We reported the main significant effects of circulating TF and YKL-40 in predicting disease relapse, indicating that an elevated YKL-40 level and TF activity demonstrate negative prognostic value. Furthermore, a worse future prognosis was shown for subjects with a combination of a higher YKL-40 concentration (>1.93 mg/mL) with higher TF activity (above 14.18 pM), since both components exert pro-tumorigenic properties. The cut-off values were established according to the median values for the whole study population. As YKL-40 is an inflammation-related molecule, its elevated level was positively associated with poor disease-free survival [14,18]. Likewise, YKL-40 controls key pathways and processes within the tumour microenvironment, including inflammation, angiogenesis, cell proliferation, differentiation, and the remodelling of the extracellular matrix and thus promotes tumour progression [15]. Interestingly, Roslind et al. claimed that serum concentrations of YKL-40 may provide a more consistent biomarker of a specific patient’s disease progression as intratumoral YKL-40 expression can vary across a single breast cancer nodule [16]. Interestingly, TF can also be secreted under pro-inflammatory conditions in a soluble form. TF can be hyperexpressed in response to TNF-α and IL-1β release [21]. Perhaps YKL-40 also stimulates TF shedding, since it is a pro-inflammatory protein. These outcomes support the concept that the inflammatory response is a complex systemic reaction and is associated with a poor outcome in patients with invasive BrC.

### 4.3. BMI’s Value as a Predictor of Disease Recurrence

Undoubtedly, an excess amount of body adipose tissue is a risk factor for breast cancer development and is associated with higher cancer mortality [3]. Surprisingly, in our study, we reported that adiposity positively influences overall survival. Similar to the results for the Kaplan–Meier plots, the Cox’s analysis showed that a low BMI (normal weight) was a predictive factor for a shorter time to disease recurrence in BrC subjects. Undeniably, this clinicopathological characteristic is crucial in disease prognosis; however, our normal-weight cohort were not affected substantially (Figure 2C). We found that patients with a BMI > 25 kg/m^2^ had a three-fold lower risk for recurrence than patients with a BMI < 25 kg/m^2^. Pajares et al. showed that obesity (BMI 30.0 to 34.9 kg/m^2^) is not associated with worse survival outcomes in operable BrC patients; only severely obese patients (BMI ≥ 35.0) had an increased risk of recurrence [6]. Additionally, Widschwendter et al. noted that severely obese subjects demonstrated significantly worse overall survival than underweight or normal-weight cases [5]. However, Playdon et al. noted that for each 5 kg/m^2^ rise in BMI, there was an approximately 18% increase in the risk of total breast cancer mortality [23]. Since the data are inconsistent, further evaluations are still required.

### 4.4. Combination of BMI Values with YKL-40 Level and BMI with Leptin and Adiponectin as Predictors of Disease Recurrence

Furthermore, our study has shown increased rates of recurrence, as well as cancer-specific mortality, in the cases with a normal BMI and a lower concentration of adiponectin as well as those with a normal BMI and a higher concentration of leptin and YKL-40, independently of other prognostic covariates. Interestingly, in our study, leptin and adiponectin did not demonstrate predictive value as single biomarkers. Thus, a normal BMI is not equivalent to proper body composition. We speculate that it can be associated with subclinical insulin resistance and hyperinsulinemia independent of adiposity, since an altered adipokine profile (high level of leptin and low level of adiponectin) leads to metabolic derangements. It follows that patients with a low adiponectin concentration develop a biologically more aggressive BrC phenotype independently of hormone receptor status [2,10], since a low serum adiponectin level is associated with elevated levels of vascular endothelial growth factor (VEGF) and insulin-like growth factor (IGF) [30]. Moreover, adiponectin exerts efficient tumour growth-limiting influence by the inhibition of the inflammasomes in breast cancer cells [10]. Adiponectin is also considered an anti-inflammatory cytokine due to its ability to inhibit nuclear factor kB (NF-kB) phosphorylation and exhibits anti-migratory activity by inhibiting the Wnt/β-catenin signalling pathway, fundamental for cancer progression [2]. However, elevated leptin levels are associated with breast cancer aggressiveness and a bad prognosis. Leptin is believed to be involved in the growth and invasion of breast cancer cells by stimulating the conversion of aromatisable androgens (androstenedione and dehydroepiandrosterone) to oestradiol [4,11]. Therefore, the diminution of adiponectin (an anti-inflammatory adipokine) and exacerbation of leptin and YKL-40 (pro-inflammatory cytokines) in normal-weight breast cancer subjects may be associated with a more aggressive tumour phenotype. Additional research is needed to further determine the exact relationship between our observed interaction and the modulation of adipokines in breast cancer.

From our study, we suggest that pre-treatment obesity itself is not the cause of cancer-associated sequelae, but the adiposity-related metabolic abnormalities include pro-inflammatory macrophage polarisation, insulin resistance, and disproportionate adipokine secretion by adipose tissue. We herein propose a tentative division of patients into metabolically healthy obese subjects and metabolically unhealthy lean individuals. It suggests a substantial heterogeneity of metabolic features in normal-weight breast cancer females. Our findings potentially reject the hypothesis that adiposity is an independent prognostic factor for developing distant metastases and death as a result of breast cancer. Apparently, obesity is a risk factor for hormone-sensitive cancer development, but it improves future outcomes. It is likely we observed a kind of “obesity paradox phenomenon” in those cases. There are data demonstrating that a moderately increased BMI ameliorates survival and the response to therapy [31]. Sánchez-Jiménez et al. suggest that increased fat mass may provide an energy depot that may be beneficial for a longer survival rate [11]. Murphy W.J. and Longo D.L. have noted a positive impact of adiposity on a better response to immunotherapy in different types of cancer [32].

According to the multivariate Cox proportional risk model and the excellent area under the ROC curve for the tested model, we have demonstrated the usefulness of applying pre-treatment YKL-40, BMI, leptin, and adiponectin levels and TF activity together as biomarkers for predicting disease relapse. Thus, our results suggest that a non-obese phenotype with high leptin and YKL-40 and low adiponectin levels in breast cancer leads to cancer cell migration and invasion to promote metastasis and reduced survival. Using the biomarkers proposed here would provide more information about future prognosis and survival rates. Leptin has been identified as a key driver in the progression of breast cancer [27]. Adiponectin was originally defined as a tumour suppressor protein by promoting the inhibition of cell proliferation and sensitisation of cells to apoptosis [27]. YKL-40 is a protein that stimulates cancer cell dissemination and progression. Adipokines could be useful diagnostic, prognostic, and predictive biomarkers, reflecting advanced-stage BrC, adverse prognosis, and an inflammatory state.

The strength of this study is that it is based on an early-stage breast cancer cohort with complete information on clinicopathological characteristics and more than four years of follow-up. In addition, the prospective design contributed to a reduction in potential biases. The major limitations of this study include the small sample size and the lack of information related to waist circumference (WC) and waist-to-hip ratio (WHR). The BMI was evaluated only at the beginning of follow-up, and further fluctuations were not examined. Additionally, the analyses in this study were conducted using a single measurement of the YKL-40, adipokines, and TF activity from one pre-treatment blood sample. The patients were recruited from a single institution, predisposing to selection and referral bias and potentially limiting the generalisability to larger populations. We excluded patients with Stage IIIA or higher and metastatic BrC tumours; perhaps this action limited the ability to assess the analysed parameters in more advanced tumours. Finally, since we only studied individuals of Polish descent, our results are, therefore, not necessarily directly applicable to other ethnic groups.

## 5. Conclusions

Our study concluded that a combination of high baseline serum levels of YKL-40 and leptin with a normal BMI as well as of a normal BMI with a lower adiponectin level confer a poor prognosis. Additionally, a single higher YKL-40 concentration and single elevated TF activity were significantly associated with poorer prognosis of breast cancer over the four-year follow-up. According to our results, we suggest that overweight breast cancer patients have a better prognosis regardless of the YKL-40, leptin, and adiponectin levels since we estimated an approximately three-fold increased risk of disease recurrence or death for normal-weight versus obese women. Further investigations that elucidate the mechanisms concerning adipose tissue derivatives, including adipokines and cytokines, may help to understand and prevent severe outcomes in normal-weight-related breast cancer.

## Figures and Tables

**Table 1:** The biomarker concentrations according to the demographic and anthropometrical determinants in the study population.

Variables/Number of Patients (%)	YKL-40 Concentration (ng/mL)	TF Activity (pM)	Leptin Concentratio*n* (ng/mL)	hsAdiponectin Concentration (ng/mL)
81 (100%)	1.93 1.43/2.83	14.18 11.59/26.92	12.58 4.70/20.64	27.05 20.63/32.10
Age				
<55 years	1.87	15.20	8.77	25.24
*n* = 41 (51%)	1.44/2.81	12.31/28.76	4.43/18.26	19.76/29.09
≥55 years	2.01	13.64	12.65	28.07
*n* = 40 (49%)	1.42/3.25	11.10/26.40	5.46/23.58	23.25/32.63
Menopausal status				
Premenopausal	1.89	17.59	7.80	25.24
*n* = 27 (33%)	1.33/2.81	12.31/25.20	4.00/19.21	19.76/30.30
Postmenopausal	1.96	13.79	12.67	27.42
*n* = 54 (67%)	1.45/2.85	11.17/29.59	5.57/22.47	22.62/32.31
Body mass index			***	I vs. III *
18.5–24.9 kg/m^2^	1.91	16.29	5.30	28.19
*n* = 38 (47%)	1.36/2.85	11.77/29.59	3.12/11.56	23.88/33.63
25.0–29.9 kg/m^2^	1.85	14.01	14.31	27.02
*n* = 28 (35%)	1.43/2.54	11.73/25.38	8.70/21.63	19.28/32.12
30.0–39.9 kg/m^2^	2.35	15.20	25.72	24.44
*n* = 15 (18%)	1.78/2.90	9.02/38.2	16.92/40.85	16.93/27.91
Number of parity	II vs. III **II vs. IV *			II vs. IV *III vs. IV *
Nulliparous	2.24	13.21	10.29	23.55
*n* = 6 (7%)	1.42/2.83	11.62/21.31	5.57/31.39	20.51/34.83
1 child	2.53	17.41	13.06	27.81
*n* = 20 (25%)	2.02/2.93	9.53/36.63	6.33/23.02	24.20/32.97
2 children	1.64	14.99	12.63	27.47
*n* = 39 (48%)	1.33/2.57	12.31/25.88	3.73/19.40	20.64/33.27
3 children	1.78	13.70	12.38	24.44
*n* = 13 (16%)	1.35/2.35	11.47/33.47	3.78/23.60	18.29/27.37
4 children *n* = 1 (1%)	1.06	11.65	9.60	12.17
5 children	1.96	15.82	10.46	21.54
*n*= 2 (2%)	1.36/2.55	8.05/23.59	4.00/16.92	14.58/28.49

Abbreviations: Significant *p*-values are highlighted: * <0.01, ** <0.001, *** *p* < 0.0001; TF—tissue factor; hsAdiponectin—high sensitive adiponectin.

**Table 2:** Baseline median and (IQR) serum levels of YKL-40, leptin, and adiponectin as well as citrate plasma TF activity stratified by Ki67, HER2 and hormone receptor expression.

Molecular Determinants/Number of Patients (%)	YKL-40 Concentration (ng/mL)	TF Activity (pM)	Leptin Concentration (ng/mL)	hsAdiponectin Concentration (ng/mL)
**Expression of Ki67**				*
<20%	1.86	13.79	12.36	28.19
*n* = 52 (64%)	1.31/2.57	10.82/25.20	4.11/18.69	22.94/33.11
≥20%	2.42	22.18	14.41	25.24
*n* = 29 (36%)	1.62/2.87	11.86/38.98	6.17/23.88	16.74/27.91
**Expression of HER2**				
Negative	1.91	14.99	12.61	27.12
*n* = 72 (89%)	1.42/2.84	11.55/29.18	4.81/20.71	20.64/32.22
Positive	2.44	12.46	8.77	26.99
*n* = 9 (11%)	1.44/2.56	11.59/15.20	3.67/16.92	19.18/27.91
**Hormone receptor status**				
ER+	2.02	14.55	12.55	27.09
*n* = 70 (86%)	1.42/2.87	11.47/26.92	4.92/20.64	20.64/32.31
ER–	1.64	14.18	13.83	27.05
*n* = 11 (14%)	1.58/2.43	11.86/31.56	3.78/22.47	14.70/28.13
PgR+	2.00	15.10	12.61	26.91
*n* = 66 (81%)	1.42/2.85	11.47/26.92	5.26/20.78	20.64/32.94
PgR–	1.78	13.70	7.79	27.13
*n* = 15 (19%)	1.58/2.83	11.77/31.56	3.67/18.26	16.74/28.13

Abbreviations: IQR—interquartile range, Ki67—proliferation marker, HER2—human epidermal growth factor receptor 2, ER—oestrogen receptor, PR—progesterone receptor, TF—tissue factor, hsAdiponectin—high sensitive adiponectin; significant *p*-values are highlighted: * <0.01.

**Table 3:** The biomarker concentrations with respect to clinical and pathological characteristics in breast cancer (BrC) subjects.

Feature/Number of Patients (%)	YKL-40 Concentration (ng/mL)	TF Activity (pM)	Leptin Concentration (ng/mL)	hsAdiponectin Concentration (ng/mL)
**Molecular subtypes**		*		I vs. IV *
Luminal A	1.91	13.92	12.61	28.88
*n* = 50 (62%)	1.33/2.92	10.61/25.20	5.57/20.64	22.62/33.63
Luminal B HER2−	2.81	36.18	12.38	25.24
*n* = 13 (16%)	1.83/2.85	19.62/39.18	4.00/19.21	20.51/27.71
Luminal B HER2+ and Non-Luminal HER2+ *n* = 9 (11%)	2.44 1.44/2.56	12.46 11.59/15.20	8.77 3.67/16.92	26.99 19.18/27.91
Basal-like	1.62	14.18	13.83	24.44
*n* = 9 (11%)	1.58/1.78	11.86/31.56	3.78/22.47	14.70/27.47
**Tumour diameter**				
T1 (2 cm)	1.87	14.99	12.38	27.13
*n* = 55 (68%)	1.42/2.92	11.62/25.88	4.43/22.47	20.64/32.31
T2 (≥2 cm <5 cm)	2.14	14.03	13.57	26.22
*n* = 26 (32%)	1.50/2.81	10.42/29.59	4.92/18.26	18.79/32.1
**Nodal status**				
LN−	2.16	16.29	12.30	27.02
*n* = 62 (77%)	1.52/2.92	11.17/25.88	4.7/22.47	20.51/32.13
LN+	1.58	13.88	13.83	27.18
*n* = 19 (23%)	1.29/2.48	11.77/29.88	4.60/18.26	23.34/31.14
**Stage of disease**				
IA	2.19	18.28	12.30	27.06
*n* = 40 (49%)	1.48/3.04	11.53/25.72	4.85/26.21	20.36/31.95
IIA + IIB	1.89	13.88	12.63	27.05
*n* = 41 (51%)	1.36/2.51	11.62/29.59	4.70/17.89	23.34/32.10
**Elston and Ellis grade**				
G1 + G2	2.03	14.06	12.51	26.83
*n* = 65 (80%)	1.43/2.92	11.47/25.88	4.92/20.64	20.64/32.13
G3	1.63	15.10	14.35	27.59
*n* = 16 (20%)	1.39/2.43	12.31/37.58	4.57/20.37	17.61/29.70
**Histological type**	*		*	**
Ductal	2.03	14.99	13.53	26.23
*n* = 69 (85%)	1.58/2.90	11.47/28.76	5.34/22.47	19.76/30.30
Lobular	1.38	13.64	5.00	32.70
*n* = 12 (15%)	1.17/2.09	12.06/24.12	2.81/13.18	28.83/34.41

Abbreviations: LN—free of lymph node involvement, LN+—lymph node involvement, TF—tissue factor, hsAdiponectin—high sensitive adiponectin; significant *p*-values are highlighted: * <0.01; ** <0.001.

**Table 4:** The multivariate Cox HRs for PFS according to the baseline serum values of BMI, TF activity, YKL-40, leptin, and adiponectin.

Variables	HR	95% CI		*p*-Value
BMI	0.75	0.59	0.95	**0.0178**
TF activity	1.07	1.01	1.14	**0.0240**
YKL-40	0.82	0.51	1.35	0.4434
Leptin	1.21	1.02	1.44	**0.0327**
hsAdiponectin	0.86	0.76	0.97	**0.0135**

Abbreviations: HR—hazard ratio, PFS—progression free survival, CI—confidence interval, BMI—body mass index, TF—tissue factor, hsAdiponectin—high sensitive adiponectin; significant *p*-values are in bold.

**Table 5:** Distribution of YKL-40, TF activity, leptin, and adiponectin in recurrent patients.

Variables/Number of Patients (%)	YKL-40 Concentration (ng/mL)	TF Activity (pM)	Leptin Concentration (ng/mL)	hsAdiponectin Concentration (ng/mL)
Progression 18.5–24.9 kg/m^2^ *n* = 8 (10%)	2.69 1.82/3.46	24.09 13.85/40.49	5.41 3.77/15.67	21.59 17.61/29.88

Abbreviations: TF—tissue factor, hsAdiponectin—high sensitive adiponectin.

## References

[1] Demark-Wahnefried W., Campbell K.L., Hayes S.C. (2012). Weight management and its role in breast cancer rehabilitation. Cancer.

[2] Gelsomino L., Naimo G.D., Catalano S., Mauro L., Andò S. (2019). The emerging role of adiponectin in female malignancies. Int. J. Mol. Sci..

[3] Picon-Ruiz M., Morata-Tarifa C., Valle-Goffin J.J., Friedman E.R., Slingerland J.M. (2017). Obesity and adverse breast cancer risk and outcome: Mechanistic insights and strategies for intervention. CA Cancer J. Clin..

[4] Ray A. (2018). Cancer and comorbidity: The role of leptin in breast cancer and associated pathologies. World J. Clin. Cases.

[5] Widschwendter P., Friedl T.W., Schwentner L., DeGregorio N., Jaeger B., Schramm A., Bekes I., Deniz M., Lato K., Weissenbacher T. (2015). The influence of obesity on survival in early, high-risk breast cancer: Results from the randomized SUCCESS A trial. Breast Cancer Res..

[6] Pajares B., Pollán M., Martín M., Mackey J.R., Lluch A., Gavila J., Vogel C., Ruiz-Borrego M., Calvo L., Pienkowski T. (2013). Obesity and survival in operable breast cancer patients treated with adjuvant anthracyclines and taxanes according to pathological subtypes: A pooled analysis. Breast Cancer Res..

[7] Rubio-Jurado B., Balderas-Peña L.M., García-Luna E.E., Zavala-Cerna M.G., Riebeling-Navarro C., Reyes P.A., Nava-Zavala A.H. (2018). Obesity, thrombotic risk, and inflammation in cancer. Adv. Clin. Chem..

[8] Chen L., Kong X., Wang Z., Wang X., Fang Y., Wang J. (2020). Pre-treatment systemic immune-inflammation index is a useful prognostic indicator in patients with breast cancer undergoing neoadjuvant chemotherapy. J. Cell Mol. Med..

[9] Breast Cancer: Statistics. https://www.cancer.net/cancer-types/breast-cancer/statistics.

[10] Pham D.V., Raut P.K., Pandit M., Chang J.H., Katila N., Choi D.Y., Jeong J.H., Park P.H. (2020). Globular adiponectin inhibits breast cancer cell growth through modulation of inflammasome activation: Critical role of Sestrin2 and AMPK signaling. Cancers (Basel).

[11] Sánchez-Jiménez F., Pérez-Pérez A., de la Cruz-Merino L., Sánchez-Margalet V. (2019). Obesity and breast cancer: Role of leptin. Front. Oncol..

[12] Gong T.T., Wu Q.J., Wang Y.L., Ma X.X. (2015). Circulating adiponectin, leptin and adiponectin-leptin ratio and endometrial cancer risk: Evidence from a meta-analysis of epidemiologic studies. Int. J. Cancer.

[13] Graf C., Ruf W. (2018). Tissue factor as a mediator of coagulation and signaling in cancer and chronic inflammation. Thromb. Res..

[14] Kjaergaard A.D., Nordestgaard B.G., Johansen J.S., Bojesen S.E. (2015). Observational and genetic plasma YKL-40 and cancer in 96,099 individuals from the general population. Int. J. Cancer.

[15] Yeo I.J., Lee C.K., Han S.B., Yun J., Hong J.T. (2019). Roles of chi tinase 3-like 1 in the development of cancer, neurodegenerative diseases, and inflammatory diseases. Pharmacol. Ther..

[16] Roslind A., Knoop A.S., Jensen M.B., Johansen J.S., Nielsen D.L., Price P.A., Balslev E. (2008). YKL-40 protein expression is not a prognostic marker in patients with primary breast cancer. Breast Cancer Res. Treat..

[17] Wang D., Zhai B., Hu F., Liu C., Zhao J., Xu J. (2012). High YKL-40 serum concentration is correlated with prognosis of Chinese patients with breast cancer. PLoS ONE.

[18] Johansen J.S., Christensen I.J., Jorgensen L.N., Olsen J., Rahr H.B., Nielsen K.T., Laurberg S., Brunner N., Nielsen H.J. (2015). Serum YKL-40 in risk assessment for colorectal cancer: A prospective study of 4,496 subjects at risk of colorectal cancer. Cancer Epidemiol. Biomark. Prev..

[19] Bielawski K., Rhone P., Bielawska S., Rość D., Brkic A., Zarychta E., Ruszkowska-Ciastek B. (2019). Heparanase link between vasculogenesis and angiogenesis as well as a predictive factor of a shorter survival rate. J. Physiol. Pharmacol..

[20] Hanahan D., Weinberg R. (2011). Hallmarks of cancer: The next generation. Cell.

[21] Christodoulatos G.S., Spyrou N., Kadillari J., Psallida S., Dalamaga M. (2019). The role of adipokines in breast cancer: Current evidence and perspectives. Curr. Obes. Rep..

[22] Arai Y., Kamide K., Hirose N. (2019). Adipokines and aging: Findings from centenarians and the very old. Front. Endocrinol. (Lausanne).

[23] Playdon M.C., Bracken M.B., Sanft T.B., Ligibel J.A., Harrigan M., Irwin M.L. (2015). Weight gain after breast cancer diagnosis and all-cause mortality: Systematic review and meta-analysis. J. Natl. Cancer Inst..

[24] Jarde T., Caldefie-Chezet F., Damez M., Mishellany F., Penault-Llorca F., Guillot J., Vasson M.P. (2008). Leptin and leptin receptor involvement in cancer development: A study on human primary breast carcinoma. Oncol. Rep..

[25] Ando S., Gelsomino L., Panza S., Giordano C., Bonofiglio D., Barone I., Catalano S. (2019). Obesity, leptin and breast cancer: Epidemiological evidence and proposed mechanisms. Cancers (Basel).

[26] Panno M.L., Naimo G.D., Spina E., Ando S., Mauro L. (2016). Different molecular signaling sustaining adiponectin action in breast cancer. Curr. Opin. Pharmacol..

[27] Maroni P. (2020). Leptin, adiponectin, and sam68 in bone metastasis from breast cancer. Int. J. Mol. Sci..

[28] Che S.P., DeLeonardis C., Shuler M.L., Stokol T. (2015). Tissue factor-expressing tumor cells can bind to immobilized recombinant tissue factor pathway inhibitor under static and shear conditions in vitro. PLoS ONE.

[29] Parise C.A., Caggiano V. (2014). Breast cancer survival defined by the ER/PR/HER2 subtypes and a surrogate classification according to tumor grade and immunohistochemical biomarkers. J. Cancer Epidemiol..

[30] Gu L., Cao C., Fu J., Li Q., Li D.H., Chen M.Y. (2018). Serum adiponectin in breast cancer: A meta-analysis. Medicine (Baltimore).

[31] Lennon H., Sperrin M., Badrick E., Renehan A.G. (2016). The obesity paradox in cancer: A review. Curr. Oncol. Rep..

[32] Murphy W.J., Longo D.L. (2019). The surprisingly positive association between obesity and cancer immunotherapy efficacy. JAMA.

